# The contribution of social norms and religious practices towards low death registration in 3 HDSS sites of Uganda

**DOI:** 10.1186/s12913-022-08589-9

**Published:** 2022-09-30

**Authors:** Gilbert Habaasa

**Affiliations:** Research and Applied Statistics Directorate, Population and Development Consult, Kampala, Uganda

**Keywords:** Social norms, Religious, Covid-19, Death registration, Practices

## Abstract

**Background:**

Uganda has low levels of death registration, estimated at two per cent by the National Identification and Registration Authority (NIRA). There are 56 tribes and over 5 religious denominations with so many social norms and religious practices that could have contributed to low death registration in Uganda. Previous studies on the factors affecting death registration have not assessed the contribution of social norms and religious practices toward low death registration in developing countries.

**Methods:**

A qualitative study design was adopted to examine the contribution of social norms and religious practices toward low death registration in the 3 Health and Demographic Surveillance systems (HDSS) sites of Uganda. The methods of data collection included: focus group discussions, key informant interviews, and a document review of the death registration booklet. 6 FGDs, 2 from each HDSS site were conducted comprising 1 female FGD of 10 participants and 1 male FGD of 10 participants. In addition, 26 key informant interviews were conducted with the district leaders, local council leaders, health care workers, cultural leaders, elderly, HDSS scouts and religious leaders in the 3 HDSS sites.

**Results:**

In the 4 sub-counties and 1 town council where the study was conducted, only 32 deaths were registered with NIRA, the Civil Registration authority in Uganda for the entire year from 1^st^ January to 31^st^ December 2020. The study shows that social norms and religious practices have contributed to the low death registration in the 3 HDSS sites in Uganda. Social norms and religious practices either hinder or discourage death registration initiatives by the government of Uganda. It was found out that burials that take place on the same day of death discourage death registration. Cultural taboo to announcing the death of infants, neonates, twins and suicides in the community hinder death registration. The burying of a woman at her parent's house after bride price payment default by the family of a husband discourages death registration. The religious institutions have their own set of rules, practices, and norms, which in most cases discourage death registration. For example, religious leaders refuse to lead funeral prayers for non-active members in religious activities. Results also showed that mixed religions in families bring about conflicts that undermine death registration. Lastly, results showed that traditionalists do not seek medical treatment in hospitals and this hinders death registration at the health facilities.

**Conclusion:**

The study shows that death registration is very low in the 3 HDSS sites in Uganda and that social norms and religious practices contribute greatly to the low death registration. To overcome the negative effects of social norms and religious practices, a social behaviour campaign is proposed. In addition, community dialogue should be conducted to identify all negative social norms and religious practices, how they are perpetuated, their effects, and how they can be renegotiated or eliminated to bring about high death registration in the 3 HDSS sites of Uganda. Lastly, there is a need for partnerships with cultural and religious leaders to sensitize community members on the effect of social norms and religious practices on low death registration in the 3 HDSS sites in Uganda.

## Background

The United Nations define death registration as the registration of every death event that occurs to all population groups in a country within a specified period [1]. Every country requires critical population data to plan for its citizens. Mortality data is one of the most important population data that is generated by law through death registration. Death registration is key in ensuring accurate monitoring and reporting of progress for countries to achieve the global sustainable development goals. Health workers and decision-makers use death registration information to understand and monitor the causes and levels of mortality in their communities [1]. Death records serve numerous administrative purposes; primary among these is the updating of other administrative databases, such as national population registers, national identification databases, voters’ register, taxation registers, and government service files [2]. In so doing, death records help to avoid identity fraud and ensure that services are correctly targeted. The Global Sustainable Development Goals (SDGs) propose an indicator of 80% death registration among countries by 2030 [1]. The World Health Statistics 2021 show that only 62% of the global 55.4 million deaths are registered through the Civil Registration system annually. Among the world regions, only 10% of deaths in Africa have been registered which is extremely lower than the rest of the world regions (Europe-98%, America-91%, Western Pacific-82%, South East Asia-61, and Eastern Mediterranean-55%) [3]. Sustainable Development Goal 3 focuses on providing healthy lives and promoting well-being for all ages. The SDG Target 3.6 aims to halve the number of global deaths and injuries from road traffic accidents by 2020 [4]. This enables countries, especially in sub-Saharan Africa, to achieve goal 3 of the SDGs by increasing death registration. In Uganda, there is no vital statistics data to monitor SDGs, and hence census and Demographic and Health Survey (DHS) are usually relied on for public health interventions and decision-making [5, 6]. However, census and DHS are usually conducted after 10 and 5 years respectively and hence may not accurately explain the actual population dynamics. An effective Civil Registration and Vital Statistics (CRVS) system with accurate vital statistics on death registration and cause of death are, therefore very paramount in meeting and monitoring the 2030 global SDGs.

The National Identification and Registrations Authority (NIRA) is mandated under the Registration of Persons Act 2015 (ROPA 2015) to register all deaths in Uganda. The business process for death registration in Uganda begins with notification at the sub-county or a health facility for the community and institutional deaths respectively. For community deaths, the sub-county chief completes death notification form 12 after receiving evidence of death from the next of kin while for institutional deaths, it is completed by the hospital administrator. A copy of the Death Notification Form 12 is then picked by the Assistant District Registration Officer (DRO) in weekly visits or when called and then entered into the Mobile Vital Records System (MVRS). It is then verified by the DRO and relayed to the National Identification Register based at NIRA Headquarters in Kampala. An informant is asked to pay UGX 5,000 for a death certificate. On presentation of payment receipt, the District Registration Officer prints under Civil Registration Information Management System (CRIMS) and issues a death certificate. NIRA estimates that about 370,000 deaths occur in Uganda annually with 61.6% and 38.4% of these deaths occurring in communities and health facilities respectively [7]. However, only about 2% of the annual deaths are registered in the Civil Registration and Vital Statistics System [7]. This implies that the majority of the deaths are never registered at sub-counties and health facilities in Uganda including in the 3 HDSS sites in Uganda.

Several studies have been conducted in both developed and developing countries to ascertain the factors affecting death registration [8–11]. Many of these studies have explored the contribution of poor laws, inadequate resources, and poor infrastructure, among others, towards low death registration, especially in developing countries. Unfortunately, limited research has been conducted to unpack the contribution of social norms and religious practices toward low death registration in developing countries. A study examining the impact of traditional and religious practices on the spread of the Ebola virus disease in West Africa revealed that some community members did not register the deaths of their relatives to Civil Registration authorities because they wanted to observe funeral traditions including washing dead bodies which were against government procedures on Ebola virus disease victims [12]. A study in South Sudan showed that the dead are buried on family land as proof of land ownership and to keep a relationship with the soil [13]. The community, therefore, do not appreciate the benefits of death registration given that burial grounds act as sufficient evidence for land ownership instead of death registration. A recent study in Uganda [9] lists over ten determinants of death registration: lack of political will, outdated legal frameworks, few registration centres, inadequate funding, uncoordinated registration systems, inadequate training among NIRA staff, lack of enforcement mechanisms, low technology use and poor attitude of registration officers among others. This study did not find out the extent to which social norms and religious practices have contributed to low death registration in Uganda.

Uganda has 56 tribes with so many social norms and religious practices that may have a far-reaching contribution to low death registration [14]. These cultural norms have been practised as a rite of passage for generations. They are further promoted by cultural institutions and their leaders in different parts of the country following the restoration of cultural institutions in 1993 after their abolition in 1967. Article 246 of the 1995 Uganda constitution provides for the revitalisation, strengthening, and support of traditional cultural institutions [14]. To date, the Government recognises and supports cultural and religious institutions that continue to propagate social norms and religious practices in Uganda. The study, therefore, examines the status of death registration and the contribution of social norms and religious practices toward low death registration in 3 HDSS sites in Uganda.

The current study, therefore, examines the status of death registration and the contribution of social norms and religious practices toward low death registration in the 3 HDSS sites in Uganda.

## Methods

### Study design and area

The study used a qualitative design to examine the contribution of social norms and religious practices toward low death registration in the 3 HDSS sites in Uganda. This design enabled the researcher to obtain in-depth information from the study respondents and participants. The study areas were: Iganga-Mayuge HDSS (IMHDSS) site in the Eastern region (Bulamagi sub-county & Nakigo sub-county), Kyamulibwa HDSS site (Kyamulibwa sub-county), and Rakai HDSS site (Kyotera sub-county & Kalisizo town council), both of which are found in Central region. These areas were chosen because the HDSS sites have monitored their populations for over ten years. The IMHDSS has a population of 85,000 people, Kyamulibwa HDSS has 22,000 people and Rakai HDSS has 50,000 people. The 3 HDSS sites are situated within two regions of Uganda with strong cultural (Busoga and Buganda kingdoms) and religious institutions (Islam, Catholicism and Protestantism religions). The 2014 Uganda National Population and Housing Census indicate that the Baganda in central Uganda and Basoga in Eastern Uganda are the biggest tribes in Uganda in the first (16.5%, 5,555,319) and third positions (8.8%, 2,960,890) respectively [5]. By religious denomination, Catholics (40%, 13,407,764) followed by Anglican (32%, 10,941,268) and Muslims (14%, 4,663,204) respectively are the biggest religions in Uganda [5]. Other Ugandans belonged to traditional religion (0.2%, 33,805) and no religion (0.1%, 78,254) [5].

### Study population and sampling

The study population included; men, women, district leaders, local council leaders, health care workers, cultural leaders, elderly, HDSS scouts and religious leaders. This population was considered because they were deemed knowledgeable about the contribution of social norms and religious practices toward low death registration in the 3 HDSS sites of Uganda.

### Sampling and data collection methods

The participants were purposively selected. The study employed Focus Group Discussions (FGDs), key informant interviews, and document reviews as data collection methods. Specifically, 6 FGDs, 2 from each HDSS site were conducted comprising of 1 female FGD of 10 participants aged 18–60 years and 1 male FGD of 10 participants in each site aged 18–60 years. The study deployed an FGD guide and the discussions lasted 1 to 2 h.

On top of FGDs, key informant interviews were conducted to obtain in-depth information from persons knowledgeable about the contribution of social norms and religious practices toward low death registration in Uganda. The study used a key informant guide to administer interviews with 26 respondents in the 3 HDSS sites in Uganda. In the Iganga-Mayuge HDSS site, 8 key informant interviews were conducted with; the sub-county chief, Local Council 1 chairperson, youth chairperson, cultural leader, Islamic religious leader, HDSS Village scout, elderly, and Village health team member. At the Kyamulibwa HDSS site, 13 key informant interviews were conducted with the assistant town clerk, sub-county chief, Imam, priest, Local Council one (LC1) chairperson, Community Development Officer, Village Health Teams (VHTs), cultural and opinion leaders. At the Rakai HDSS site, 5 key informant interviews were conducted with the Senior town clerk, LC1 chairman, religious leader, Senior Hospital administrator, and a nurse. On average, each Key informant Interview lasted 40 to 60 min. Lastly, a document review was used to extract the number of deaths from the death registration booklet from 1^st^  January to 31^st^ December 2020 at the four sub-counties and one town council in the study area. A document review checklist was utilised to gather the necessary data to inform the study. During data collection, the participants' voices from focus group discussions and key informant interviews were audio-recorded by the research assistants and later transcribed verbatim. The researcher read through all transcripts and identified themes and subthemes to guide the analysis.

The study was anchored on the social norms theory [15] and the theory of planned behaviour [16] to examine the contribution of social norms and religious practices toward low death registration in 3 HDSS sites in Uganda. Social norms refer to unwritten rules governing the behaviour of people living in the community [15]. The existence of social norms in the community motivates the actions of people and can even exert pressure on individuals to act in a particular way due to the positive or negative consequences accruing to them [15]. The theory of planned behaviour on the other hand explains how religious practices demotivate individuals to register deaths of their loved ones with the Civil registration authorities [16].

### Data analysis and reporting

All the transcripts were imported into ATLAS.ti software and were assigned to two coders who worked independently on each transcript. The coded transcripts by each coder were uploaded to the Qualitative Data Analysis Programme to establish the intercoder reliability (ICR) by computing Kappa scores. The Kappa scores for the first 10 coded transcripts were recorded. All the inconsistencies between the two coders were discussed and an agreement on the final codes was arrived at which improved the Kappa scores to between 0.65 to 0.80. Coding was conducted using deductive and inductive coding approaches. This means that higher-order codes or themes were generated from the research questions while the sub-themes were derived from the responses of the study respondents and participants. Finally, all the analysed data was used to inform reporting and manuscript preparation.

## Results

### Status of death registration in HDSS sites of Uganda

In the 4 sub-counties and 1 town council where the study was conducted, only 32 deaths were registered with NIRA, the Civil Registration authority in Uganda for the entire year from 1^st^ January to 31^st^ December 2020. By location, death registration is equally low: Bulamagi (2 deaths), Nakigo (6 deaths), Kyamulibwa (11 deaths), Kyotera (3 deaths), and Kalisizo Town council (10 deaths). The findings from the qualitative study in all the 3 HDSS sites confirm a low prevalence of death registration as elaborated below:

a *“Death registration was prominent in the 1980s. By then, it was a must that people had to report to the sub-county chiefs. But people nowadays do not register deaths at the sub-county offices due to various reasons, including distance, cultural norms, money, and religion. In the past month, we have buried many people, but we have not registered any.*
b (KII, Kyamulibwa Sub-county, Kyamulibwa HDSS).

The few death registrations recorded at the sub-counties are usually done more than three months after a death. This translates into delayed and late registration according to the ROPA 2015. This is further elaborated by one of the key informants thus:

a *“When a person dies, people do not immediately come to register the death. They come three months later or many years later to register a death especially to process powers of administration for the estate of the deceased. The death registration process is initiated by the family members by asking for a death confirmation letter from the chairperson of the Village to be brought to the sub-county chief. I confirm that the only time people come for death registration is when they are going to the administrator general”.*
b (KII, Nakigo Sub-county, Iganga-Mayuge HDSS).

### Social norms and their contribution toward low death registration

#### Burials on the same day or 1–2 days after death diminish the opportunities for death registration

Adults are usually buried after 3–4 days of death as the family awaits children, relatives, and community members to gather. On the other hand, the burial of young people usually takes place on the same day or after 1–2 days of death. Adults are persons aged above 18 years while young people are those aged below 18 years as per the constitution of Uganda. Adults usually have properties that are distributed amongst the children of the deceased. Hence, local leaders are typically notified about the deaths because they are usually involved in affirming the property distribution of the dead. When a death occurs, community members are not expected to engage in economic activities such as cultivation until burial is completed. All community members gather at the deceased home and mourn the dead until burial takes place.

When the burial is completed on the same day of death, the majority of the people including the family are then preoccupied with their day-to-day chores and activities thereby diminishing the chances of reporting such deaths to civil registration authorities. The above findings are further elaborated thus:

a *“When an adult dies, we do not go to the garden that day but instead congregate with the family of the deceased, days before and after the burial to comfort them. Many people attend the burial, and they come from as far as the towns. However, for young people or children, few people attend. No one can even think of registering such a death, yet adults can be registered”.*
b (Participant, FGD with men, Bulamagi Sub-county, Iganga-Mayuge HDSS).

#### Customary wills and heir installation hinders death registration

Findings show that customary wills and installation of the heir after the death of a family head do not require the processing of letters of administration or even a death certificate. The fact that clan or community members believe in the will read and implemented during “Kwabya olumbe,” i.e., the last funeral rite demotivates the family members to register the death with the civil registration authority. The community and family members follow the customary will in the distribution of the deceased’s estate to the beneficiaries. Similarly, the heir installed is given powers to manage the affairs of the deceased’s family as elaborated thus:

a *“When the head of a family who has been sick for some time dies, he leaves a will with someone who is not from his family. This ‘will’ is supposed to be read on the burial day or some days after, giving inheritance details of the deceased and mentioning the heir. The heir is allocated a piece of land and sometimes a stool as a symbol that he has taken over leadership from the deceased. This gives the heir recognition and authority over the property of the deceased. Therefore, there is no need to register the death of the deceased to obtain powers of administration for the property since everyone acknowledges the heir installed after this ritual locally known as okwabya olumbe.”.*
b (Participant, FGD with men, Kyotera Sub-county, Rakai HDSS).

#### Cultural taboo against the announcement of death for an infant or neonates hinder death registration

Cultural taboos hinder family members from death announcements for infants and neonates in the community and this cuts across the 3 HDSS sites where the study was conducted. The civil registration authorities and the community members are never notified about these deaths. Infants and neonates are not announced that they have died and are usually buried by family members only. If it is a girl, they cover her in a banana plant known as “Nakitembe” and a boy is buried under sour banana plantain called “mbidde.” Findings from the elderly in the HDSS community revealed that people are not informed and only women who have stopped giving birth attend burials for infants and neonates. This is due to the fear of transmitting a bad omen to young women of reproductive ages. This hinders such deaths from being registered with the Civil Registration Authorities thus:

a *“We do not make death announcements for infants and neonates. A few close family members will get to know it because it is considered a cultural taboo and is looked at as insignificant. Only older women who have stopped giving birth are allowed to attend the burial. Women who are still giving birth are not allowed to bury. This makes death registration unlikely for such a death.”.*
b (Elderly, KII, Kyotera Sub-county, Rakai HDSS).

#### Bride price payment default discourages death registration

Payment of bride price influences death registration among the Baganda and the Basoga tribes in the study area. When a married woman dies before the bride price has been paid by the husband's family, the husband's family is forced to pay the bride price before burial. Else, the body is taken and buried at the home of the woman’s parents. A social norm of burying a woman at her parent's home after the bride price payment default brings shame to the woman’s parents in their community and consequently discourages death registration. Besides, the parents find no reason to get bothered with the death registration of their daughter with the civil registration authorities. This is clarified by some of the respondents who cited that married women whose bride price had not been paid by the time of death were buried at their parent's home, a practice that hindered death registration:

a *“If a married woman dies before the bride price has been paid, she has to be buried at her parents’ home. This is a very painful and shameful experience for the parents of the deceased. Many parents become remorseful and choose not to engage in activities that remind them of the death of their daughter. They don’t want to think of their children as dead, which death registration confirms”.*
b (Participant, FGD with women, Kyotera sub-county, Rakai HDSS).

#### The cultural taboo to reporting the death of a twin hinders death registration

Deaths of twins have low chances of being registered by the civil registration authorities due to cultural norms regarding such deaths as mere “jumping” or “disappearance” of a twin in the study area. Given that twins share a lot in common, the word “death of a twin" is avoided so that the surviving twin is not scared that he or she will die. Further, when twins die, tradition requires them to be buried at night and not passed through the doors of the house but rather a hole is dug where the bodies are passed before taking them to the burial grounds. This level of concealment diminishes the chances of death registration for twins. Given that it is culturally unacceptable to say that a twin has died, the parents are not interested to register a death of a twin or even associating with the word ‘death of a twin” to avoid negative consequences to the living co-twin. This is elaborated in the key informant interview thus:

a *“In this community, people do not say that a twin has died; they call it ‘kubuuka,’ which means that a twin has jumped. When someone says that a twin has died, the other twin can also die since they were produced together. Some say that he has gone to collect firewood. There is no way people can go to the sub-county chief and notify him that a twin has died. It would be opposing what we believe in”.*
b (VHT, KII, Kyamulibwa Sub-county, Kyamulibwa HDSS).

#### Suicide deaths are buried in secrecy and this limits death registration

Suicide deaths are viewed as a curse to a family and can spread for generations as per the culture of the Baganda and Basoga tribes in the 3 HDSS sites of Uganda. Hence, such deaths are seldom registered with the civil registration authorities. Based on their culture, the residents of Bulamagi Sub-county, Iganga-Mayuge HDSS site consider life precious and believe that a person who commits suicide brings a curse to the family and has to be punished in a manner that prevents others from doing the same. Consequently, no family member is willing to register a suicide death with the Civil Registration Authorities. These views were expressed by a cultural leader saying:

a *“Suicide is a terrible thing in our culture. This is because someone has brought a curse into the family that can spread to all family members for generations. The deceased's body is beaten, and the rope used for strangulation is cut so that the body falls into a hole dug directly below. It is buried in whatever position it falls in, even if it is crooked. People are not supposed to arrange it because that is what the deceased wanted for him or herself. People are not supposed to mourn such a death. Few people attend burials of people who have committed suicide. Their family members attend because they have no choice. No one can register such a death”.*
b (Cultural Leader, KII, Bulamagi Sub-county, Iganga-Mayuge HDSS).

### Religious practices and their contribution toward low death registration

#### Religious leaders’ refusal to lead funeral prayers undermines death registration

Some religious institutions and leaders refuse to lead funeral prayers for the dead, contributing to low death registration in the communities. Community members that are not involved in church or mosque activities, including paying tithe, and attending prayers, often fail to have religious leaders pray for them when they die or their close relatives. These findings suggest that death registration status should not be conferred on those who deviate from the model of personhood–in this case, as articulated by the Church or mosque. This makes them unworthy of death registration with Civil Registration Authority. A key informant mentioned that when priests fail to conduct funeral prayers for people who do not follow the rules put in place by Catholic Church, it increases grief and psychological torture to the family to the extent of foregoing death registration with the Civil Registration Authorities thus:

a *“I should say we do not conduct mass for people who act contrary to our faith. For instance, couples who die before being officially married in the church as well as converts from Catholicism to other religions like Islam and born-agains. It increases grieving and is psychologically torturing for family members when mass is not conducted. They can forego death registration because they feel that the deceased has not been decently put to rest. People will think that the deceased's spirit is still roaming on the earth and fear to refer to them as dead”.*
b (Religious Leader, KII, Nakigo Sub-county, Iganga-Mayuge HDSS).

#### Mixed religions in families bring conflicts that hinder death registration

Mixed religions in families bring about conflicts that undermine death registration. For example, in the Nakigo sub-county of Iganga district, parents refused to attend the burial of their children because they had defected from Islam. In addition, the parents of the dead were unwilling to register the dead with the civil registration authority due to the rejection of their children, as elaborated thus:

a *“Family members can refuse to attend the burial of their children because of religious conflicts. For instance, a girl might be a Muslim and later becomes a Christian. The parents can even refuse to attend her burial and cannot register her death if they are the next of kin. This is because they did not approve religious conversion”.*
b (Participant, Female FGD, Nakigo Sub-county, Iganga-Mayuge HDSS).

#### Traditionalists seek medical treatment in shrines and witch doctors which undermines death registration

The study found out that persons who believed in traditional religions did not seek medical treatment in hospitals and therefore resorted to traditional medicine in shrines and from witch doctors. Besides, traditionalists regularly attribute the cause of death to sorcery which hinders death registration and the medical examination of the cause of death. Even if someone dies of a motor accident, they will attribute the death to sorcery or ghosts. This consequently hinders death registration with the Civil Registration Authorities which is associated with post-mortem that is usually done in health facilities to establish the cause of death before registering the death. In light of the above, the study findings noted that a substantial proportion of the population takes sick people to traditionalists who perform rituals and later send them back home. In case of death of the sick at home, fewer efforts are put in place to register the dead with the Civil Registration authorities as elaborated thus:

a *“In cases where people die from the hospital, the family knows the cause of death. Some people who are not taken to hospital are taken to shrines where traditionalists tell them the root cause of the problem, perform rituals and send them back home. When they die at home, the family members are aware of the cause of death based on what the traditionalists told them. So, they just go ahead to bury the deceased. However, this decreases deaths at the hospitals where causes of death can be investigated, and the hospital staff fills out death notification forms before the discharge of the deceased as part of the death registration process”.*
b (Assistant Town clerk, KII, Kyamulibwa Sub-county, Kyamulibwa HDSS).

#### The catholic church registration of the dead is mistaken for official death registration

The study noted that the Catholic churches are fond of registering the dead and maintaining a death register for their members in the areas of jurisdiction. Findings revealed that religious registration of the dead by the Catholic church was mistaken with the official death registration by Civil Registration Authorities. As a result, some community members had not registered their dead with Civil Registration Authorities because the catholic church took records of their dead to pray for them and for administrative purposes. These records are never relayed to the Government’s Civil Registration Authorities which is perhaps a missed opportunity in death registration initiatives for Uganda. The catholic church uses catechists who record particulars of the dead and then feed them into the death registration book for the church. This scenario is elaborated below:

a *“The catholic church has religious heads in every parish locally known as the ‘Eitwale’ who register the death of every catholic that regularly attends mass. They have forms that record the death details, including the cause of death. A person who registers the death of their family member with the Eitwale cannot register with the sub-county chief because it is like double registration and wastage of time”.*
b (FGD Participant for women, Bulamagi Sub-county, Iganga-Mayuge HDSS).

## Discussion

This study describes the status of death registration and the contribution of social norms and religious practices toward low death registration in 3 HDSS sites in Uganda. Low death registration in the Iganga-Mayuge, Kyamulibwa, and Rakai HDSS sites of Uganda is consistent with many other areas, especially in developing countries. From the year 2000 to 2015, a moderate increase in death registration from 36 to 38% is reported globally [17]. Death registration is strongest in Europe, America, Australasia and a few countries in East Asia and Latin America. The least performance in death registration is reported especially in Sub-Saharan Africa [17].

The study findings showed cultural taboos against announcing death for infants, neonates, twins and even suicides have hindered death registration in 3 HDSS of Uganda. The findings are consistent with a study in Nigeria in which low death registration was reported among new-borns in the south-south region of Nigeria [18]. The deceased's parents did not register newborn deaths because the babies had lived for only a short time. The deaths of the young are not registered with civil registration authorities by the relatives because it reminds them of the “deceased’s short unfulfilled life” [19]. In addition, superstitious beliefs among the Yoruba and Igbo in Southern Nigeria term some children as “Abiku or Ogbanje”. These children are said to belong to a group of spirits with an unfulfilled purpose on earth. When these children finally die, their communities are expected never to discuss or recognize that they died [19]. Given the kind of discrimination against the children termed as “Abiku” and “Ogbanje” by their communities and families, the chances of reporting their deaths to Civil Registration Authorities are usually slim.

The study findings revealed that bride price payment default discouraged death registration in the 3 HDSS sites in Uganda. Consistent with the findings, a study in Egypt revealed that death registration was lower among women whose parents did not receive the bride price than their counterparts with parents that received the bride price [20]. This agrees with the current study findings in which failure to pay the bride price by the bridegroom denied his family an opportunity to bury his wife. This consequently discouraged the deceased’s family from registering the death with the civil registration authority.

The study found that customary wills are recognised as adequate in handling inheritance and property rights in the 3 HDSS sites of Uganda. The recognition of customary wills in property rights as well as in installing the heir with full family headship rights poses a significant challenge to death registration. The fact that clan or community members believe in the will read and implemented during the last funeral rights “Kwabya olumbe” does not motivate the family members to register the death and obtain a death certificate since the customary will called “*Ekilamo”* is sufficient. The above findings are contrary to studies that recognised formal and legal wills requiring a land title as the only mode of settling inheritance and land rights [21].

On the contribution of religious practices toward low death registration, the current study shows that mixed marriages in families undermined death registration due to religious conflicts. This is consistent with the findings in a study conducted in Norway in which religious intolerance was observed in the 19^th^ and twentieth centuries [22]. Religious fanatics could not associate with the dead who had converted from their traditional religions including registering such deaths.

The study findings also reveal that traditional believers who seek medical treatment in shrines and witch doctors undermined death registration. The above findings are also consistent with a South African study in which 6% of all respondents to verbal autopsy interviews attributed the death of women and children to witchcraft [21]. Even relatives of people who died without seeking hospital-based health care attributed witchcraft as the cause of death. In addition, a study in Guinea Bissau noted low levels of death registration in the communities (2%) in which witchcraft was mentioned as a key determinant [10].

Although the findings place social norms and religious practices as contributing factors to low death registration, some studies suggest a contributory role of CRVS supply and demand-side challenges. On the supply side, death notification is currently done at sub-counties and health facilities which is costly in terms of time and funds to travel from a village to the sub-county headquarters since they are far apart by a big distance [7]. In Nigeria, the proximity to a civil registration office increased the likelihood of death registration by the next of kin [19]. The Registration of Person’s Act 2015 in Uganda is not fully enforced. For example, although death registration is compulsory, no effort to establish the status of registration by local authorities before burials take place in communities. Death registration is mostly paper-based and sometimes registration materials are not distributed to registration centres on time. In addition, the levying of fees on death notification forms by sub-county local governments and health facilities discourage death registration. Lastly, the COVID-19 pandemic travel restrictions and the total lockdown instituted by the president of Uganda between March 18^th^ to June 4^th^ 2021 could have limited death registration.

On the demand side, the National CRVS Strategy for Uganda 2020/21–2024/25 indicates inadequate awareness about death registration including benefits, processes and requirements [7]. Although death registration serves legal, administrative and statistical purposes [1], most community members are not aware or cannot easily identify with its existing incentives. For example, although death registration provides documentary evidence of identity for claiming benefits of the property, insurance and remarriage among others, a section of the public does not need death registration to inherit property. The majority of the dead are employed in informal businesses and subsistence agriculture where insurance packages are not available. In addition, polygamy is practised and recognized under customary marriage and hence cases of death registration of a spouse as an incentive for remarriage are minimal.

The previous studies done in the HDSS sites of Uganda showcase mortality underreporting [23, 24]. For instance, the Iganga-Mayuge HDSS study revealed cases of mortality underreporting [23]. The study response rate over years was affected by vacant households (10.4%), demolished households (5.8%), changes in the status of residence (5.5%), and absence from home (0.2%) as well refusals of 0.04%. The underreporting of deaths is very prevalent in low-income countries, especially in Sub-Saharan Africa where about 1% of deaths are registered [19]. In addition to the above, Nahuche HDSS in Zamfara State of North-West Nigeria with similar settings as those of the 3 HDSS sites in Uganda where the study was conducted shows high mortality underreporting especially among the 0–4 year age group [25]. This was due to cultural taboos in death reporting, especially among neonates and infants.

The current study provides a clear understanding of how social norms and religious practices contribute to low death registration in Uganda and possibly other developing countries. The study presents additional knowledge that can be expounded through future research. However, considering that death is a very personal experience, the study did not conduct in-depth interviews with men and women to find out the reasons for low death registration. In addition, the 6 FGDs and 26 KIIs may not necessarily represent the views of all persons in the 3 HDSS sites hence presenting a sample limitation. There is also a possibility that some other factors such as transport costs to the registration centres could have contributed to low death registrations on top of the social norms and religious practices as found out in this study.

## Conclusion

Based on the study findings, there are few deaths registered with the Civil registration authority in the 3 HDSS sites in Uganda. The analysis results show that the social norms and religious practices have a significant contribution to the low death registration in the study area. To change and overcome the rigid effects of social norms and religious practices, a social behaviour campaign should be put in place by NIRA, district leaders, non-governmental organisations and other stakeholders operating in the 3 HDSS sites of Uganda. Community dialogues should be conducted to identify all negative social norms and religious practices, how they are perpetuated, their effects, and how they can be renegotiated or eliminated to bring about high death registration in the 3 HDSS sites in Uganda. The dialogues can be facilitated by the local leaders and other interested stakeholders. Lastly, there is a need for partnerships with cultural and religious leaders to sensitize community members on the effect of social norms and religious practices on low death registration in the 3 HDSS sites in Uganda.

## Data Availability

All data generated or analysed during this study are included in this published article.

## Abbreviations

COVID-19: Corona Virus Disease
CRIMS: Civil Registration Information Management System
CRVS: Civil Registration and Vital Statistics
DHS: Demographic and Health Survey
DRO: District Registration Officer
FGDs: Focus Group Discussions
HDSS: Health and Demographic Surveillance System
HIV: Human Immunodeficiency Virus
IDRC: International Development Research Centre
IMHDSS: Iganga-Mayuge Health and Demographic Surveillance System
IRB: Internal Review Board
IUSSP: International Union for the Scientific Study of Population
KII: Key Informant Interview
LC1: Local Council one
LG: Local Government
MUSPH: Makerere University School of Public Health
MVRS: Mobile Vital Records System
NIRA: National Identification and Registration Authority
ROPA: Registration of Persons Act
SDGs: Sustainable Development Goals
UNCST: Uganda National Council of Science and Technology
UNICEF: United Nations Children’s Fund
VHTs: Village Health Teams
